# Does Performing Endoscopy Sooner Have an Impact on Outcomes in Patients With Acute Nonvariceal Upper Gastrointestinal Hemorrhage? A Systematic Review

**DOI:** 10.7759/cureus.16092

**Published:** 2021-07-01

**Authors:** Zahid Ijaz Tarar, Muhammad Usman Zafar, Umer Farooq, Ghulam Ghous, Hafiz Muhammad Hassan Shoukat, Vanessa Kuwajima

**Affiliations:** 1 Internal Medicine, University of Missouri, Columbia, USA; 2 Hospital Medicine, Lehigh Valley Health Network, Allentown, USA; 3 Internal Medicine, Loyola Medicine, MacNeal Hospital, Berwyn, USA; 4 Hematology/Oncology, University of Missouri, Columbia, USA; 5 Internal Medicine, Premier Health/Wright State University, Dayton, USA; 6 Gastroenterology and Hepatology, University of Missouri, Columbia, USA

**Keywords:** nonvariceal hemorrhage, urgent upper endoscopy, esophagogastroduodenoscopy (egd), hemostasis, mortality

## Abstract

Background

Endoscopy is the cornerstone for the diagnosis and treatment of nonvariceal upper gastrointestinal bleeding. Regarding the management of nonvariceal bleeding, the administration of crystalloid solution and proton pump inhibitors before endoscopy is well established, but the optimal timing of endoscopy has been a matter of debate and a subject of many investigational studies. The need for urgent endoscopy arises to provide prompt redress to acute bleeding, decrease the length of stay, and lower mortality from ongoing bleeding.

Objective

This study aimed to determine if endoscopy performed within 24 hours of presentation improves outcomes in terms of mortality, hospital length of stay, and rebleeding in individuals presenting with nonvariceal upper gastrointestinal bleed with any risk.

Methodology

We performed a systematic review of two large databases (PubMed and Google Scholar) to incorporate all studies published after 2000. We included studies with nonvariceal upper gastrointestinal bleeding and excluded those reporting variceal gastrointestinal hemorrhage.

Results

We reviewed eight studies that qualified after meeting our inclusion and exclusion criteria. We divided these studies into three separate groups based on the timing of endoscopy. Only two studies found a difference in mortality that was statistically significant in patients who underwent endoscopy within 24 hours of presentation. One study showed lower mortality in a patient who underwent urgent endoscopy, but it did not reach statistical significance. Other studies did not show any statistical difference in mortality, hospital length of stay, and rebleeding rates. The studies showed conflicting evidence on the amount of blood transfusion, though urgent endoscopy was found to be difficult in few studies due to blood obscuring the lesion.

Conclusions

While data suggest that there is a potential benefit in performing endoscopy sooner, there is no concrete evidence to point to a particular time range. Before performing endoscopy, the American Society for Gastrointestinal Endoscopy (2012) recommends adequate resuscitation with crystalloid solutions, blood transfusions, and antisecretory and prokinetic agent therapy. More investigational studies are needed to formulate a time-sensitive flow sheet to approach endoscopy in patients with nonvariceal upper gastrointestinal bleeding. A strict criterion is also needed to delineate patients into low-risk and high-risk groups. Doing so would provide a systematic approach to help with mortality, rebleeding, and healthcare resource utilization.

## Introduction

Nonvariceal upper gastrointestinal bleeding is one of the most common gastrointestinal tract problems with a significant impact on the healthcare system, averaging 300,000 hospitalizations yearly with a total expenditure of 7.6 billion US dollars. Endoscopy is the cornerstone for the diagnosis and treatment of nonvariceal bleeding. Advancements in endoscopy techniques are proving beneficial in decreasing mortality [1-3]. Endoscopy can identify the cause of bleeding in 80% of patients, yet mortality from peptic ulcer disease, which is the most common cause of upper gastrointestinal bleeding, ranges between 5% and 14%. This incidence is further increased in the elderly with multiple comorbidities [4-6].

The optimal timing for endoscopy has been a matter of debate and a subject of many investigational studies. Recently, several authors have tried to tackle this question and determine whether patients need to be risk-stratified to assess the appropriate timing. Although there is significant evidence that endoscopy performed within 24 hours of presentation is beneficial in terms of mortality and rebleeding, there is limited data to show how soon endoscopy should be performed [7,8]. Results from different studies yield conflicting results; for example, in studies performed by Cho et al. and Lim et al., decreased mortality was found, but in similar studies conducted by Tai et al. and Targownik et al., no significant difference was seen [9-12]. It is to be noted that the population size was larger in the former studies which could be one reason for a significant difference.

The need for urgent endoscopy arises to provide prompt redress to acute bleeding, decrease the length of hospital stay, and lower mortality from ongoing bleeding. The decision to perform endoscopy could be deferred in individuals with low-risk features, including age <60 years, those who do not have major comorbidity, and are hemodynamically stable. Therefore, a criterion to stratify patients is needed. Some scores have been developed and are useful in these instances. Two very well-known and validated scores are the Glasgow-Blatchford score (GBS) and Rockall score which help in categorizing patients into low-risk and high-risk groups [6,13]. Of these, GBS is considered superior. By dividing patients into low-risk and high-risk groups, we can identify patients who would require endoscopy sooner. In an ideal setting, patients who undergo prompt endoscopy with identification and treatment of the culprit lesion and are hemodynamically stable should be discharged with outpatient monitoring. Hospital length of stay and resource utilization would be affected by this. However, Bjorkman et al. were unable to find any difference in resource utilization [14]. The theoretical benefits of urgent esophagogastroduodenoscopy (EGD) in providing source identification and control, characterization of the lesion, and need for hospital admission exist but concrete evidence is limited. In studies conducted by Laine et al. and Kodali et al., it was highlighted that patients with clean ulcers were at low risk for recurrent bleeding and could be potentially managed in an outpatient setting. This should help in resource utilization in the long term; however, provider behavior might be variable with regards to admitting or discharging low-risk patients [15,16]. In Bjorkman et al., the decision to admit a patient after endoscopy was taken by the admitting provider and not the endoscopists. The admitting providers continued to admit patients despite being advised of the low risk of particular patients [14].

Theoretically, if bleeding lesions are identified and treated earlier, one would hypothesize that the risk of rebleeding would decrease and that there would be a mortality benefit and associated decrease in the length of hospital stay, cost, and resource utilization. However, as is evident above, this has not been the case in a majority of the studies reviewed.

## Materials and methods

**Figure 1:**
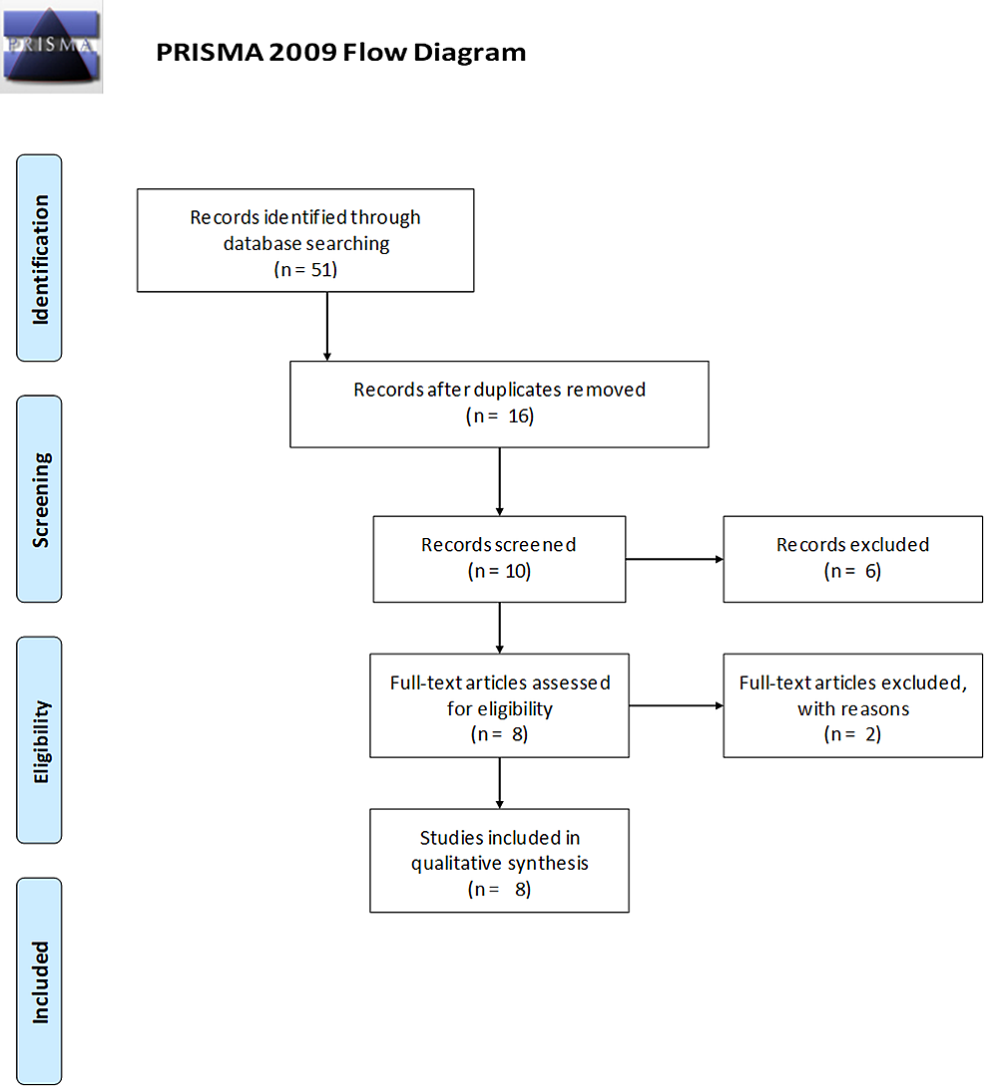
PRISMA flowchart describing study selection and inclusion process. PRISMA: Preferred Reporting Items for Systematic Reviews and Meta-Analyses

We performed a literature search on two large public databases, namely, PubMed and Google Scholar, to identify studies on the topic published after 2000. The following search words were used to identify studies from Google Scholar: gastrointestinal bleeding, timing, endoscopy, nonvariceal, early, and delayed. A total of 2,770 search results were obtained. A meticulous search was performed and studies meeting the following exclusion criteria were not selected: (i) not in English, (ii) articles on variceal bleeding, (iii) Articles involving complications of portal hypertension, (iv) articles on upper gastrointestinal hemorrhage that did not compare early versus delayed nonvariceal bleeding, (v) review articles, and (vi) articles with clear intervals not defined. Articles were reviewed by both reviewers and disagreements were resolved by consensus. A total of 17 articles were identified in this manner from Google Scholar and 34 articles from PubMed. After further discussion, articles with a mention of variceal bleeding and no further comments on time intervals were excluded. After accounting for duplicates, eight studies were identified for review. These studies were reviewed for the effectiveness of early endoscopy, primary outcomes on the patient population, and economic effects from different time intervals (Figure 1).

## Results

After selecting articles for review, the studies were divided according to the time limit set for endoscopy. By doing this, we wanted to analyze and compare studies that evaluated the same time period against each other. We discuss the studies below.

Table 1 summarizes two studies that compared endoscopies done within eight hours of presentation to the ones done 8-24 hours of presentation. In the first study, Tai et al. compared outcomes for patients who presented to the emergency department with nonvariceal gastrointestinal bleeding and were deemed to be at high risk. This was a retrospective study that identified 189 high-risk individuals (88 received endoscopy within eight hours and the rest 101 within 8-24 hours) who presented to the emergency department (ED) within a six-month time interval (July 2004 to December 2004). High-risk individuals were defined as age more than 60 years, severe comorbidity, active bleeding (witnessed hematemesis, red blood per nasogastric tube, hematochezia), hypotension or shock, red blood cell transfusion of more than six units, and severe coagulopathy. The study had clearly defined inclusion and exclusion criteria. The comparison duration of eight hours was selected because working hours were eight hours per day. This study did not find any difference in transfusion rate, length of hospitalization, and mortality rate in both groups. The most commonly found lesions on endoscopy were duodenal and gastric ulcers but without any statistical difference between the two groups. Although the mortality rate was lower in the group undergoing endoscopy within eight hours (1% vs, 6%), the data did not reach statistical significance, even though more actively bleeding lesions were detected. One shortcoming in this study was that among the group that underwent endoscopy within eight hours patients were found to retain more blood. This makes EGD more difficult to complete, puts an individual at risk for bronchoaspiration, and necessitates repeat EGD. Additionally, in this study, more patients underwent combined endoscopic therapeutic modalities in the early intervention group without any significant effect on outcomes.

The second study was carried out by Ahn et al. (Table 1). In contrast with the above study, this study examined outcomes from urgent EGDs done “after hours” [17]. Data were obtained from procedures performed between January 2009 to December 2010 in a tertiary hospital in Korea by two experienced endoscopists. A total of 158 patients were included in the study based on admission timing “after hours,” and out of these, 60 received endoscopy in eight hours and the rest 98 within 8-24 hours. The time limit for the urgent EGD was set within eight hours from the initial admission. There was no difference in primary outcomes among the two groups, which was defined as primary hemostasis, recurrent bleeding, and 30-day mortality. There was no statistically significant difference in secondary outcomes defined as hospital length of stay (although the length of hospital stay was higher in patients who underwent EGD within 8-24 hours), transfusion requirements, and recurrent endoscopies. Similar to Tai et al., the most frequently found bleeding lesion in this study was peptic ulcers. Patients in this study were also followed up two months post-discharge and had no recurrence of bleeding in either group. In a multiple linear regression analysis, they found that urgent EGD (within eight hours) would decrease the hospital length of stay. The strength of this study is that it chose to address the effect of urgent EGD after hours when staffing can be an issue. Moreover, it followed patients two months post-discharge to evaluate any immediate adverse events. A shortcoming, however, was that it did not have clearly defined inclusion/exclusion criteria, although the patients were stratified using the Rockall score into low-risk and high-risk groups. In addition, although the study put great emphasis on using experienced endoscopists for better outcomes, they were unable to compare outcomes against inexperienced endoscopists. Both these studies looked at a smaller subset of patients, and maybe a larger sample size would find some difference. Both studies did not address the role of proton pump inhibitor (PPI) on endoscopic outcomes.

**Table 1:** Comparison of outcomes in endoscopy performed within eight hours versus within 8-24 hours. EGD: esophagogastroduodenoscopy

Source	Patients	Design	Outcome measured	Conclusion
Tai et al. (2007) [11]	189; high-risk patients	Retrospective	Mortality rate, length of stay, total amount of transfusion, rate of recurrent bleeding	No statistical difference regarding the rate of recurrent bleeding, total amount of transfusion, length of stay, and mortality rate in both groups
Ahn et al. (2016) [17]	158; all risk	Retrospective	Mortality and hospital length of stay	Clinical outcomes were not significantly different between the urgent and early EGD groups

Table 2 lists studies that compared endoscopies performed within six hours of presentation against those done 6-48 hours after presentation. A total of three studies were identified. The largest of these was conducted in Korea by Cho et al. The study included 961 patients that were deemed to be high risk with nonvariceal bleeding. This retrospective observational study examined patients with a GBS greater than 7 who presented to their emergency department. The data spanned a period of nine years. Compared to patients who underwent endoscopy within six hours (urgent) versus those who underwent endoscopy within 48 hours (delayed), the study found significant differences in mortality rate, the number of transfused packed red blood cells, need for intervention, and embolization among the study groups. The mortality rate was higher among the delayed group. The need for transfusion, intervention, and embolization was higher among the urgent group. The study found no difference in rebleeding, intensive care unit admission, vasopressor use, and length of stay. The study had strict inclusion/exclusion criteria. Variceal bleeding and tumor bleeding were excluded. All patients were initiated on PPI. This study had several strengths including strong inclusion and exclusion criteria, patients with GBS of >7 in the high-risk group compared to previous studies that only included patients with GBS of >12, and it found increased mortality among patients with malignancy and cirrhosis independently. Limitations of this study included that it did not compare endoscopies on low-risk patients, patients were observed only at one hospital, and it included patients with malignancy and cirrhosis that had the potential to skew data.

Bjorkman et al. (Table 2) aimed to observe outcomes of patients with nonvariceal upper gastrointestinal bleeding in clinical settings where non-gastroenterologists were decision-makers. This was done in lieu of a highly structured study because patients presenting in a real-world setting may not be evaluated by a strict criterion. This study was a prospective, randomized, blinded, and multicenter trial that compared patients undergoing endoscopy within six hours (urgent) versus those undergoing within 48 hours (elective). The study did not include patients considered to be high risk, as determined by hemodynamic instability and a Rockall score of 6 or 7. Data were analyzed with the intention to treat. The study did not find any difference in primary outcomes defined as healthcare resource utilization (length of stay, intensive care unit days, blood transfusions, and the need for additional interventions) or patient outcomes. It is to be noted that the study was terminated early because an interim analysis showed that continuing the study would not alter the primary outcomes so only 93 outpatients were studied. However, because mortality rate and recurrent bleeding were secondary outcomes, it was not determined if extending the study would have affected the outcomes. A strength of this study is that the disposition of the patient was determined by the attending and not the investigator endoscopist which allowed to obtain real-life data analysis. The admitting provider chose to admit patients despite being labeled as low risk. It can be debated that the outcomes would have been different if the patients were discharged instead, but this decision was allowed by the study design.

Targownik et al. (Table 2) reviewed patients who presented to two different tertiary care centers in Canada with acute nonvariceal upper gastrointestinal bleeding between 1999 and 2004. The inclusion criteria delineated 169 patients into two groups, in which one group underwent endoscopy within six hours (rapid) and the other had endoscopy within 24 hours (early). The study was limited to individuals with hemodynamic instability as defined by systolic blood pressure <100 mmHg and heart rate >100 beats per minute. Data from the rapid group reached statistical significance for having more active bleeding, nonbleeding visible vessels, or an adherent clot when compared to the early group. Patients undergoing urgent endoscopy were less likely to receive PPI which could affect the study because it has been shown that prior use of PPI can improve visualization of lesions. This study failed to identify any statistical significance in primary outcomes (25% rapid group versus 23% early group; P > 0.2) such as in-hospital rebleeding, in-hospital mortality, any surgical intervention, and 30-day readmission. Although a decrease in the likelihood of developing adverse gastrointestinal outcomes was seen in the rapid endoscopy group, this was not statistically significant (odds ratio [OR]: 1.16; 95% confidence interval [CI]: 0.53-2.52). Like other studies in the ongoing discussion, this study was limited by sample size as well. Additional shortcomings were a lack of structured PPI use and a lack of controls.

**Table 2:** Comparison of outcomes in endoscopy performed within six hours versus within 6-48 hours. ICU: intensive care unit; UGIB: upper gastrointestinal bleeding; ANVUGIB: acute nonvariceal upper gastrointestinal bleeding

Source	Patients	Design	Outcome measured	Conclusion
Cho et al. (2018) [9]	961; high risk	Retrospective	Mortality and rebleeding within 28 days of admission	Urgent endoscopy was an independent predictor of lower mortality rate and was not associated with rebleeding in high-risk patients with acute nonvariceal UGIB
Bjorkman et al. (2004) [14]	93; all risk	Randomized prospective clinical trial	30-day resource utilization, including the number of hospital days (total length of stay), ICU days. Blood transfusion, and the need for additional intervention (surgery, endoscopy, radiology). Secondary outcomes included the frequency of recurrent bleeding, mortality rate, and other morbidities	Urgent endoscopy did not reduce hospitalization or resource utilization
Targownik et al. (2007) [12]	169; high risk	Retrospective	Development of any adverse bleeding outcome (rebleeding, surgery for control of bleeding, in-hospital mortality, or readmission within 30 days for ANVUGIB	No significant difference in adverse bleeding outcomes

Table 3 summarizes the three studies that compared endoscopies done within 24 hours against those performed within 12-13 hours. Lim et al. studied 934 patients over a period of 18 months prospectively and divided them into low- and high-risk groups based on a GBS of 12. All patients with a GBS less than 12 were considered low risk and those above 12 were included in the high-risk group. Of the 934 patients, 97 met the criteria for high risk, and the rest 837 were low risk. The primary outcome measured in this study was all-cause in-hospital mortality. The study compared outcomes in low- and high-risk patients who received EGD in 13 hours versus after 13 hours. Inclusion and exclusion criteria were clearly defined and all patients with variceal bleeding and lower gastrointestinal bleed confirmed on colonoscopy were excluded. The study found that high-risk patients required more endoscopic treatment, blood transfusion, ICU admission, and rebleeding episodes compared with low-risk patients. In high-risk patients who underwent endoscopy after 13 hours, all-cause mortality was very high (44% to 0%), with a statistical significance of <0.001, and these patients all had decrease length of stay without a large difference in blood transfusion needs, rebleeding rates, or subsequent surgery. On the other hand, in low-risk patients, endoscopy after 13 hours was not associated with an increase in all-cause mortality. Additionally, the authors performed a subgroup analysis on 725 patients who received EGD with 24 hours and after 24 hours due to various reasons to determine that if GBS of 12 and above still yield similar results if EGD is done within 13 hours. This subgroup analysis verified the previous results of decreased mortality in high-risk patients who received EGD within 13 hours compared to those who underwent EGD after 13 hours (0% vs 48%) with a P-value of <0.001. The analysis also confirmed that there was no difference in mortality in low-risk groups based on EGD timings. An unfortunate drawback to this study was that the sample size of high-risk versus low-risk was small potentially adding bias to the study.

Saleem et al. (Table 3) conducted a retrospective observational study of patients who frequented a tertiary hospital in upstate New York in a year between January and December with an upper gastrointestinal bleed. Of the 806 charts reviewed by the authors, 251 patients were included in the analysis. These patients were divided into three groups: those who received EGD in <12 hours of admission (urgent endoscopy), 12-24 hours of admission (early endoscopy), and >24 hours after admission (late endoscopy). Similar to the discussion above, the authors found significant differences in the presence of blood obscuring the scope and timing of EGD. This was one of the reasons why patients in the urgent EGD group underwent a second EGD. No differences in length of hospital stay, the number of units of blood, and mortality were identified with respect to the timing of EGD. The upside to this study was that the patients had similar risk based on the Blatchford scores and no difference in mortality and hospital length of stay was found. This review was limited by its retrospective nature.

The study by Jairath et al. (Table 3) is one of the largest studies in this review that included 212 UK hospitals studying 4,478 patients. Data were collected for two months on patients with acute upper gastrointestinal bleeding in these centers. Similar to Saleem et al., patients were grouped into three groups based on endoscopy performed within 12 hours, 12-24 hours, or >24 hours. No difference in mortality was seen between those who underwent endoscopy early (<12 hours) and those examined later (12-24 hours, OR: 0.99, 95% CI: 0.97-1.02; or >24 hours OR: 0.98, 95% CI: 0.88-1.09; P = 0.7). Patients who underwent endoscopy earlier experienced rebleeding more than the other two groups (19.7% vs. 10.9% vs. 8.8%, respectively; unadjusted ORs for rebleeding 0.75, 95% CI 0.69-0.81 at 12-24 hours; 0.63, 95% CI 0.56-0.72 at >24 hours; P < 0.001). However, after adjusting for confounding variables, no difference was seen. This study demonstrated a reduction in risk-adjusted length of stay and found that early endoscopy led to improved control of hemorrhage in high-risk patients. One of the strong points of this study was that it used a mixed-effects logistic regression model to examine the relationship between time to endoscopy and mortality to adjust for confounding variables.

**Table 3:** Comparison of outcomes in endoscopy performed within 13 hours versus within 13-24 hours.

Source	Patients	Design	Outcomes measured	Conclusion
Lim et al. (2011) [10]	934; all risk	Prospective	All-cause in-hospital mortality	Lower mortality in high-risk but not in low-risk patients
Saleem et al. (2020) [21]	250; all risk	Retrospective	Mortality, hospital length of stay, or number of blood transfusions received, surgical or interventional radiology-guided interventions	No difference in mortality, number of units of blood transfused, or length of hospitalization
Jairath et al. (2012) [25]	4,478; all risk	Randomized control trial	Mortality, rebleeding, need for surgery, and length of hospital stay	No reduction in mortality or need for surgery. However, there was increased efficiency of acre and potentially improved control of hemorrhage in high-risk patients

## Discussion

Endoscopy is the most effective method for diagnosing and treating nonvariceal gastrointestinal bleeding [18]. Current guidelines recommend performing endoscopy within 24 hours in high-risk patients who are hemodynamically unstable, have a hemoglobin of <8 g/dL, or present with shock. For patients with suspected variceal bleed, however, guidelines recommend performing endoscopy within 12 hours [19]. No such recommendations exist for bleeding due to causes other than variceal hemorrhage. Various studies have been performed to study the benefit for patients undergoing endoscopy for nonvariceal hemorrhage within 24 hours of admission. Our review focused on studies that ascertained the benefit of endoscopy in nonvariceal hemorrhage sooner than 24 hours.

The only studies that showed mortality benefit in this review were Cho et al. and Lim et al. [9,10]. In the study by Tai et al., the mortality rate was lower in the group that received endoscopy sooner; however, this data did not reach statistical significance [11]. In the study by Cho et al., the focus was rightfully set on high-risk individuals, and while the study did find mortality benefits, it is interesting to know that the patients who received endoscopy sooner also were transfused larger blood volumes and were more likely to receive intervention than the delayed group. While this could potentially skew data, there was no difference in rebleeding. Lim et al. have been criticized for including a small sample of high-risk patients and doubt has been shed on the study’s design. Having a larger sample size gives the study by Cho et al. a potential advantage. In a study done on more than one million Medicare beneficiaries undergoing endoscopy, a mortality benefit was likely found due to the large sample size [20].

Before performing endoscopy, the American Society for Gastrointestinal Endoscopy (2012) recommends adequate resuscitation with crystalloid solutions and blood transfusion in hemodynamically unstable patients followed by antisecretory and prokinetic agent therapy. Some of the studies above did not have a strict criterion for PPI use before endoscopy. In these studies, it was observed that in patients who underwent endoscopy sooner, more active bleeding was found, and it was more likely that in these patients the bleeding obscured the primary lesion [11,12,21]. Previous data have shown that gastric visualization improves with agents introduced prior to endoscopy like PPI or prokinetic agents [22-24].

Lim et al. and Jairath et al. noticed differences in rebleeding rates in the groups that underwent endoscopy sooner [10,25]. Overall, no difference was found among other studies. As mentioned by Cho et al., rebleeding was understandably more evident in patients with high-risk lesions, and these patients were more likely to undergo endoscopy sooner [9]. While not all studies had structured PPI use as mentioned above, studies that did use PPI argue that the time between PPI use and urgent EGD might be short enough that minimal healing occurred. This might have prompted findings of more active bleeding and/or increased the risk of rebleeding.

Resource utilization and hospital length of stay can be directly affected by the timing of endoscopy [26]. If we can identify patients who have had a gastrointestinal bleed but are at low risk, they could potentially be discharged from the hospital with outpatient follow-up. Bjorkman et al. set out to study these parameters. However, they were unable to identify any difference in healthcare utilization by performing endoscopy earlier [14]. The decision to admit patients after endoscopy was delegated to admitting providers, and despite being informed about the low risk, these patients were still admitted. More studies are needed to accurately predict these parameters and to instill confidence in decision-makers to send stable patients home with outpatient follow-up.

There are some limitations of our review. This review includes mostly retrospective studies, and more randomized controlled trials are needed to elucidate differences in the timing of endoscopy. Additionally, studies characterized subjects into high-risk and low-risk based on different criteria, although there were several similar risk factors. Our review could also be limited by publication bias where one seeks to publish favorable outcomes. However, we have followed PRISMA guidelines for systematic review and discussed relevant points from all studies after agreement from authors. While we have limited evidence suggesting mortality benefit from endoscopy performed sooner than 24 hours, more work needs to be done to identify patients that would benefit from this procedure, a clear definition of early endoscopy, appropriate guidelines to recommend discharge for patients at low risk, and utilization of these recommendations for better healthcare cost and utilization.

## Conclusions

Even though the data is suggestive of potential benefit in performing endoscopy sooner, there is no concrete evidence to point to a particular time range. With regards to the management of nonvariceal bleeding, the following aspects are clear. On arrival, the patient should have intravenous access and receive crystalloid solutions and PPI. While we have limited evidence suggesting mortality benefit from endoscopy performed earlier than 24 hours, more work needs to be done to identify patients who would benefit from this procedure, a clear definition of early endoscopy, appropriate guidelines to recommend discharge for patients at low risk, and utilization of these recommendations for better healthcare cost and utilization. More large center prospective studies are needed to formulate a time-sensitive flowsheet to approach endoscopy in patients with nonvariceal upper gastrointestinal bleeding.
